# Methyl-CpG binding domain protein acts to regulate the repair of cyclobutane pyrimidine dimers on rice DNA

**DOI:** 10.1038/srep34569

**Published:** 2016-10-03

**Authors:** Changxun Fang, Weisi Chen, Chengxun Li, Xin Jian, Yingzhe Li, Hongmei Lin, Wenxiong Lin

**Affiliations:** 1 Fujian Provincial Key Laboratory of Agroecological Processing and Safety Monitoring, College of Life Sciences, Fujian Agriculture and Forestry University, Fuzhou 35002, P. R. China; 2 Key Laboratory of Ministry of Education for Genetics, Breeding and Multiple Utilization of Crops, Fujian Agriculture and Forestry University, Fuzhou 350002, Fujian, P. R. China; 3 Key Laboratory of Crop Ecology and Molecular Physiology of Fujian Universities, Fujian Agriculture and Forestry University, Fuzhou 35002, P. R. China

## Abstract

UVB radiation causes cyclobutane pyrimidine dimers (CPDs) to form on the DNA of living organisms. This study found that overexpression of the silicon absorbance gene *Lsi1* reduced the accumulation of CPDs in rice, which profited from the reactivation by photolyase. The transcript abundance of *deoxyribodipyrimidine photolyase (Os10g0167600*) was generally correlated with the silicon content of the rice, and the up-regulation of *Os10g0167600* was found to be highest in the UVB-treated *Lsi1*-overexpressed (*Lsi1*-OX) rice. A trans-acting factor, methyl-CpG binding domain protein (*Os*MeCP), was found to interact with the cis-element of *Os10g0167600*. The nucleic location of *Os*MeCP effectively enabled the transcriptional regulation. Compared with the WT, the level of *Os*MeCP was lower in the *Lsi1*-OX rice but higher in the *Lsi1*-RNAi line. Rice cultured in a high silicate-concentration solution also exhibited less *Os*MeCP abundance. Overexpression of *OsMeCP* led to lower *Os10g0167600* transcript levels and a higher CPD content than in the WT, but the reverse was true in the *OsMeCP*-RNAi line. These findings indicate that *Os*MeCP acts as a negative regulator of silicon, and can mediate the repression of the transcription from *Os10g0167600*, which inhibits the photoreactivation of the photolyase involved in the repair of CPDs.

Because the highly active components of radiation penetrate cells, solar ultraviolet B (UVB) radiation (280 to 315 nm) generally plays a negative role in plant growth and development by causing oxidative damage and cross-links in plant cells^1^. Lesions form in nuclear, chloroplast, and mitochondrial DNA due to the accumulation of cyclobutane pyrimidine dimers (CPDs) and pyrimidine (6-4) pyrimidone photoproducts (6-4 PPs) on the DNA strands, which have been recognized as two major lesions on DNA^2^,^3^.

To maintain genomic integrity, repairing DNA damage is essential for an organism to survive^4^. Studies show that plant photoreactivation plays an essential role in repairing the CPD damage caused by UVB radiation, and that photolyase participates in this pathway by absorbing blue/UVA (320 to 400 nm) light and using the energy to monomerize the dimers^5^ in a process known as the photorepair pathway. In addition to photorepair, dark repair occurs in plants, including excision repair for bases or nucleotides, mismatch repair, and other DNA repair pathways. Photorepair and excision repair are both important for maintaining the stability of the genome and are essential for an organism’s survival. DNA damage repair and its relative photolyases have been extensively reported in several plant species^6^,^7^,^8^,^9^,^10^.

There are four genes that encode photolyases in rice, namely *Os10g0167600, Os02g0204400, Os03g0343400*, and *Os09g0532700*, and three genes that encode the photoreceptor, namely, *Os02g0625000*, *Os02g0573200*, and *Os06g0661800*. These genes are located in different parts of the chromosome and their expression can be activated and increased in the presence of UVA and blue light to increase photoreactivation for repairing DNA damage^11^,^12^. Overexpression of *CPD photolyase* in both UVB-sensitive and UVB-hypersensitive rice contributes to increased CPD photolyase activity, which increases the plants’ resistance to growth damage from UVB compared with wild-type (WT) plants^13^. The activity of CPD photolyase in rice can be regarded as a symbolic factor for evaluating the sensitivity of rice to UVB radiation^13^,^14^.

Beneficial elements such as silicon have been reported to contribute to the enhancement of UVB resistance in rice^15^,^16^. The ability of rice to absorb silicon from the environment is controlled by the gene that encodes the NOD26-like major intrinsic protein; Ma *et al.* found the gene in a low-silicon rice mutant and named it *Lsi1*^17^. Fang *et al.* inhibited and overexpressed *Lsi1* on rice to generate two types of rice with different silicon contents, and compared their gene expression profiles after exposure to UVB. The *CPD photolyase* gene expression was up-regulated in the transformed rice line with *Lsi1*-overexpression (*Lsi1*-OX) but down-regulated in the *Lsi1*-RNAi line, and these results were comparable to those for the WT. It has been suggested that the *CPD photolyase* gene expression was correlated with the silicon content in rice and that silicon could activate the expression of photolyase^18^. However, the underlying mechanism is still unknown.

We conducted a comparative study of the expression of *photolyase* encoded genes to select the responsible gene in types of rice with different silicon contents. The most correlative *photolyase* genes triggered by silicon were obtained and the positive trans-acting factor interaction with the cis-acting element of the responsible *photolyase* gene was revealed. The bio-interaction of the trans-acting factor with silicon and photolyase was then further investigated to illuminate the regulation pathway for repairing the CPDs on rice DNA.

## Results

### Silicon, CPD, and 6-4 PPs contents in the Lemont rice accession with *Lsi1*-OX, *Lsi1*-RNAi, and WT

**Figure 1:**
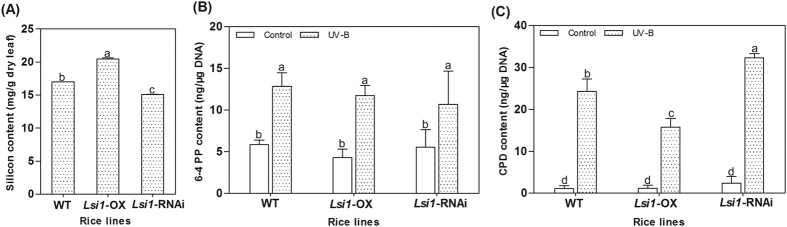
Silicon, 6-4 PP, and CPD contents in the leaves of the *Lsi1*-OX and *Lsi1*-RNAi transformed lines, and the WT Lemont rice accession. (**A**) The silicon content in the leaves of the three test rice lines under normal growth conditions; (**B**) the 6-4 PP content in the leaves of the three test rice lines for the UVB-treated group and the control group; (**C**) the CPD content in the leaves of the three test rice lines for the UVB-treated group and the control group. WT, wild type of Lemont rice accession; *Lsi1*-OX, *Lsi1* overexpression transformed Lemont; *Lsi1*-RNAi, *Lsi1* RNA interference transformed Lemont. Data are represented as the mean ± SEM. The different superscripts in the columns indicate statistical groups that are significantly different (p < 0.05) using Tukey’s range test.

Overexpression of *Lsi1* in the Lemont rice accession (*Lsi1*-OX) resulted in increased silicon content in the leaves, and the silicon content in the *Lsi1*-RNAi transformed Lemont was notably lower than that in the WT (Fig. 1A). Exposing the rice to UVB radiation led to increased CPD and 6-4 PP contents in the leaf DNA. There was no obvious difference in the 6-4 PPs contents between the transformed rice and the WT under the same light conditions (Fig. 1B). In contrast, the CPD content was significantly lower in the leaves of the *Lsi1*-OX line than in the WT after exposure to UVB radiation. A 35.17% decrease was found in the *Lsi1*-OX line compared with the WT. However, a notable increase of 33.16% was found in the *Lsi1*-RNAi line compared with the WT (Fig. 1C). These results indicate that the increased *Lsi1* expression in the rice enhanced the silicon content in the leaves and reduced the CPD content in the DNA of the leaves under UVB irradiation, while inhibition of *Lsi1* expression in the rice samples led to the opposite result.

### *Photolyase* gene expression and the *photoreceptor* in the Lemont rice accession with *Lsi1*-OX, *Lsi1*-RNAi, and WT

Based on our transcriptome sequencing data from the *Lsi1*-OX and *Lsi1*-RNAi transformed Lemont rice lines and the WT rice, which were irradiated with UVB for 5h, and the control group (full data not shown here), we found seven genes that encoded photolyase or the photoreceptor, and which were differentially expressed in the three UVB-treated rice lines and the control condition. Among these genes, the transcription of *Os10g0167600* was up-regulated when the rice samples were exposed to UVB. Changes of 1.51, 2.59, and 1.57 times were detected in the WT, *Lsi1*-OX, and *Lsi1*-RNAi lines, respectively, when comparing the UVB group to the control group. The gene encodes deoxyribodipyrimidine photolyase to repair the CPDs on the UVB-damaged DNA. Similarly, up-regulation of the *Os03g0343400* gene expression increased by 1.57, 1.12, and 1.31 times in the three rice lines, and the gene encoded a putative photolyase/blue-light receptor PHR2. In contrast, the transcript abundance of *Os09g0532700*, which is encoded as the deoxyribodipyrimidine photolyase protein-like family, was down-regulated in all three UVB-treated rice lines. *Os02g0204400* gene expression was slightly down-regulated in the UVB-treated WT and *Lsi1*-RNAi transformed lines, but it was obviously up-regulated in the *Lsi1*-OX transformed line. *Os02g0204400* encodes the (6-4) DNA photolyase that catalyzes the photoreactivation of the 6-4 PPs.

**Figure 2:**
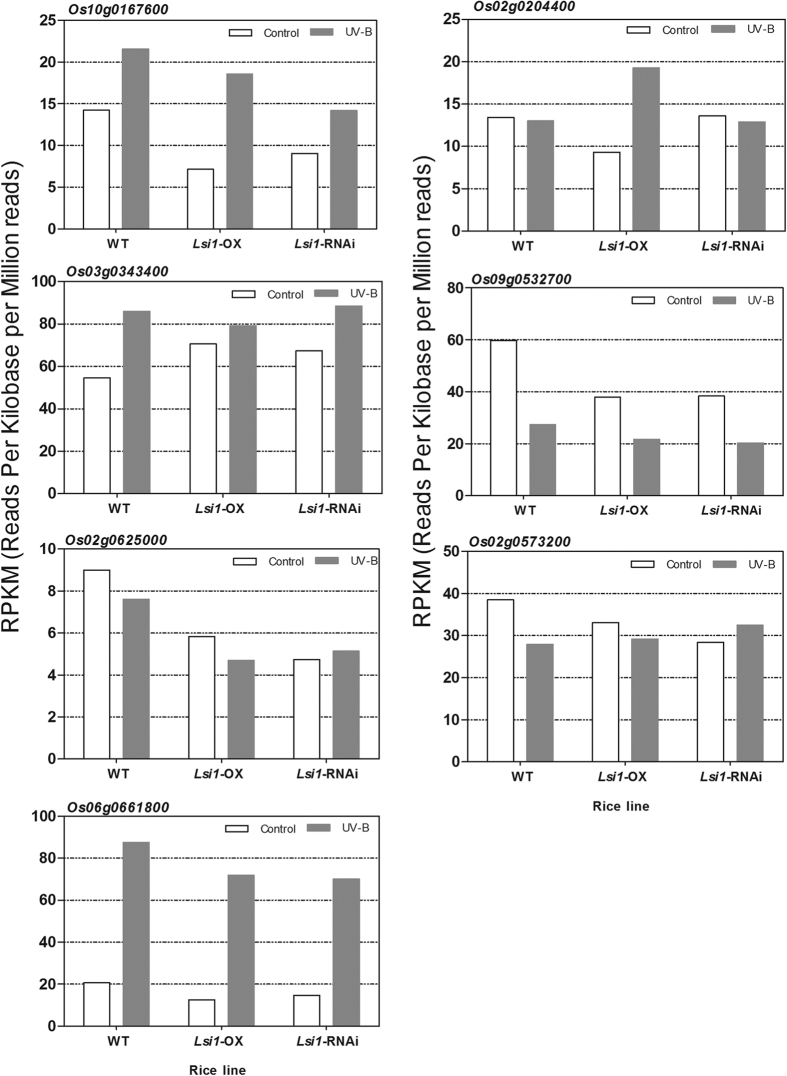
Transcriptional abundance of seven genes encoding photolyase or the photoreceptor in the UVB-treated *Lsi1*-OX, -RNAi, and WT lines of Lemont and the control groups. The transcriptional abundance of the seven genes was calculated as the reads per kilobase per million reads (RPKM), which is a method of quantifying gene expression from RNA sequencing data by normalizing the total read length and the number of sequencing reads. The RNA sequencing data were generated from the mRNA of the UVB-treated *Lsi1*-OX, -RNAi, and WT lines, respectively, and the control groups. The full RNA sequencing data are not shown here.

The other three genes also exhibited changes in their levels of expression. *Os06g0661800* was notably up-regulated in all three rice lines, as it is encoded as cryptochrome with blue-light photoreceptor activity but lacks photolyase activity. In contrast, the transcription levels of *Os02g0625000* and *Os02g0573200* were down-regulated in the UVB-treated WT and *Lsi1*-OX lines, but were slightly up-regulated in the UVB-treated *Lsi1*-RNAi rice (Fig. 2). Comparison of the changes in the transcript levels of the genes suggests that *Os10g0167600* is the most correlative to silicon in rice and plays a role in the photoactivation of CPDs on damaged DNA.

### Gene dynamic expression of *Os10g0167600* in the *Lsi1*-OX, -RNAi transformed Lemont, and WT lines

**Figure 3:**
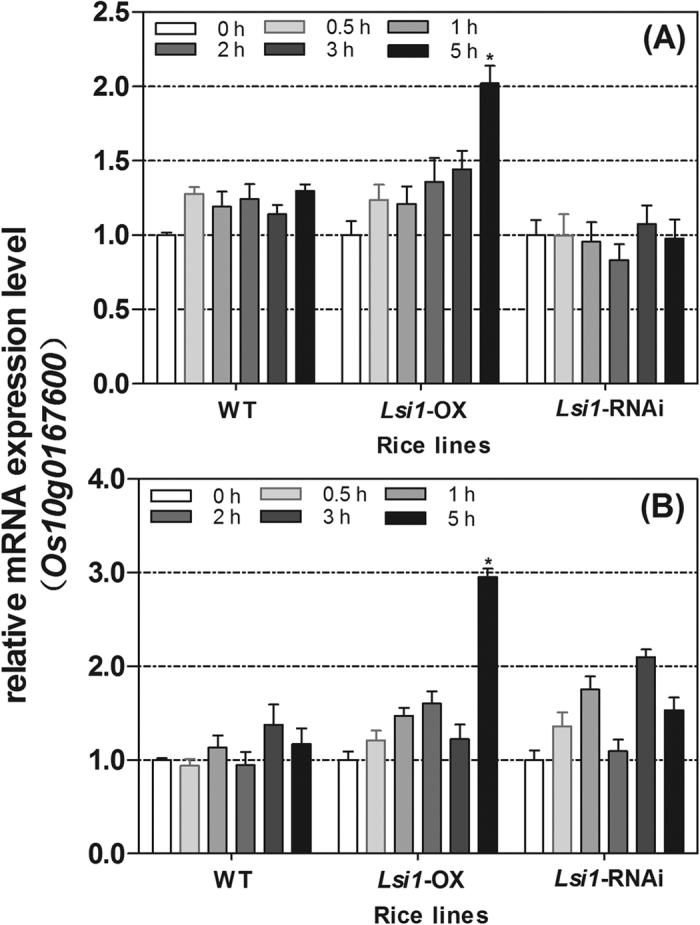
Dynamic change in the relative mRNA expression levels of *Os10g0167600* gene in the *Lsi1*-OX, -RNAi, and WT lines of Lemont under UVB radiation and the controls. (**A**) Gene dynamic expression of *Os10g0167600* in the rice lines treated by UVB radiation for 0.5, 1, 2, 3, and 5 h compared to that of 0 h (before UVB treatment). (**B**) Gene dynamic expression of *Os10g0167600* in the rice samples treated by UVB radiation for 0.5, 1, 2, 3, and 5 h compared to the control group with the same levels of irradiation. The gene expression level of *Os10g0167600* before UVB treatment (0 h) was normalized as a value of 1. Relative mRNA expression level >1 and expression level <1 indicate the comparative up-regulation and down-regulation, respectively, of the gene expression in the UVB treatment and control samples. Data are represented as the mean ± SEM. Asterisks on the columns of 5 h UV-B treated *Lsi1*-OX indicate groups that are statistically significantly different (p < 0.05) using Tukey’s range test.

qPCR was used to detect the gene dynamic expression of *Os10g0167600* in the leaves of rice exposed to UVB irradiation for 0.5, 1, 2, 3, and 5 hours. Compared with the expression of *Os10g0167600* in the rice samples before UVB treatment, UVB irradiation resulted in the up-regulation of *Os10g0167600* gene expression in the *Lsi1*-OX transformed and WT lines, and the level of up-regulation increased with time. The increase in the transcription level was higher in the *Lsi1*-OX transformed line than in the WT and peaked at 5 h of irradiation, at which point there was a 2.02 times increase in the UVB-irradiated *Lsi1*-OX transformed line compared to its control before treatment. However, no obvious change in *Os10g0167600* gene expression was found in the *Lsi1*-RNAi transformed line after UVB treatment (Fig. 3A). Compared with the control group under the same exposure time, the gene expression was also found to be up-regulated in the UVB-treated group, with a 2.95 times increase in up-regulation in the UVB-treated *Lsi1*-OX transformed line (Fig. 3B). The dynamic change in *Os10g0167600* gene expression detected by qPCR confirmed the results of the RNA-seq. Based on these results, *Os10g0167600* was selected for further study.

### Promoter of *Os10g0167600* and the interacted trans-acting factor

**Figure 4:**
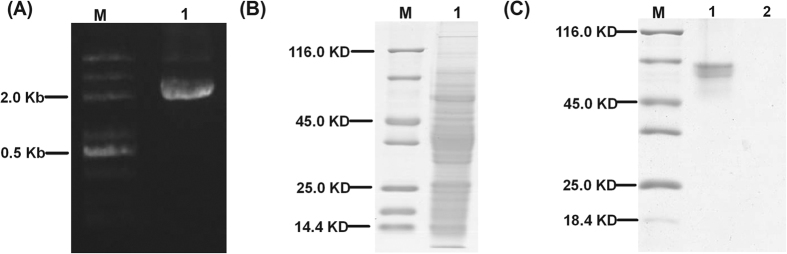
DNA-pull down screening of the interacting proteins on the promoter of *Os10g0167600.* The biotin-labeled DNA fragment of the promoter of *Os10g0167600* was mixed with the leaf total natural protein to catch the interacted proteins. The mixture was immobilized with streptavidin-coupled beads and then eluted with 1 M NaCl and forwarded to SDS-PAGE. (**A**) gene fragment of the promoter of *Os10g0167600*, M, 100 bp DNA ladder, lane 1, promoter of *Os10g0167600*; (**B**) the leaf total natural proteins of Lemont, M, protein ladder, lane 1, leaf total natural proteins; (**C**) the interacted protein on the promoter of *Os10g0167600.* M, protein ladder, lane 1 shows the proteins interacting with the DNA fragment of the promoter of *Os10g0167600*, lane 2 shows the negative control in which the DNA fragment is mixed with the protein extracting solution.

A 2154-bp fragment from the upstream region of the 5′ UTR of *Os10g0167600* was amplified from Lemont (Fig. 4A) and then incubated with the natural protein (Fig. 4B) to reveal the interacting trans-acting factor according to the DNA pull-down method (Fig. 4C). Based on the results, an obvious protein band was obtained with SDS-PAGE separation. Nine proteins (see Table S2) were identified by LC-MS. Methyl-CpG binding domain containing protein (*Os*MeCP) was regarded as the putative interacting trans-acting factor (Table 1).

### Subcellular localization of *Os*MeCP in rice

**Figure 5:**
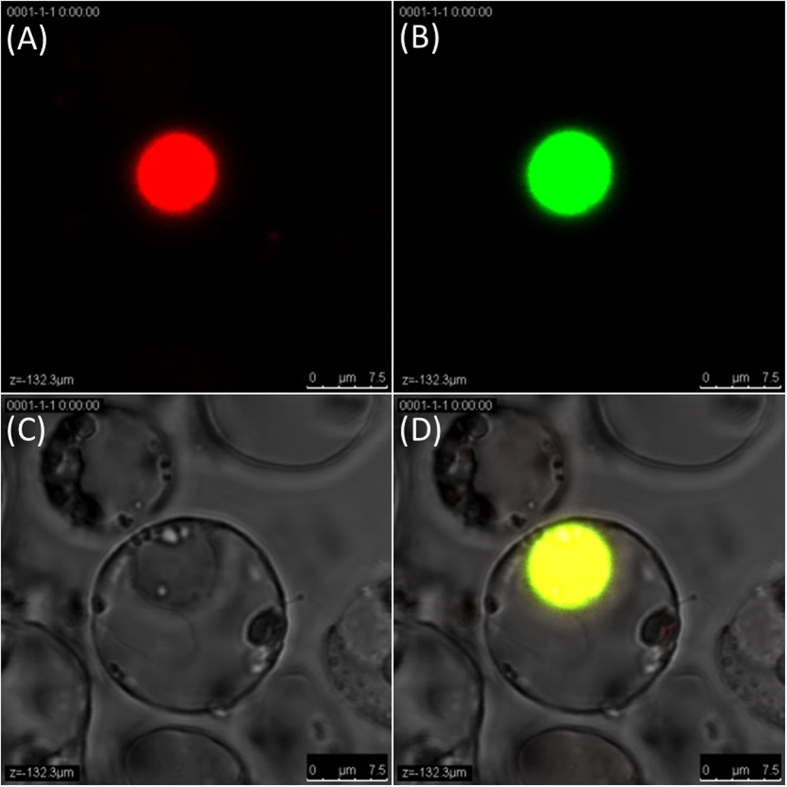
Fluorescent and bright field visualization of *Os*MeCP subcellular localization on the rice protoplast. The fluorescence images were acquired at 568-nm excitation and 592-nm emission using an Olympus Fluoview FV1000 laser scanning confocal microscope. Image (**A**) is a monomeric far-red fluorescent protein TagFP635 (scientific name mKate) that tagged the nuclear localization signal protein localized to the nucleus, and was taken as the standard marker to indicate the location of the nucleus in the rice protoplast. Image (**B**) is a fluorescent field visualization of *Os*MeCP and image (**C**) is the bright field visualization of the rice protoplast. Image (**D**) is the overlay of images (**B**,**C**) to clearly show the *Os*MeCP subcellular localization.

It is well known that gene regulation usually occurs in the nucleus and that the trans-acting factor acts in the nucleus. As indicated by the nuclear marker tag (Fig. 5A), the nucleic subcellular localization of *Os*MeCP also supports this deduction. The laser confocal imaging revealed *Os*MeCP with high fluorescence in the nucleus (Fig. 5B–D).

### Gene transcript levels of *OsMeCP* in the *Lsi1*-OX, -RNAi transformed Lemont, and WT lines

**Figure 6:**
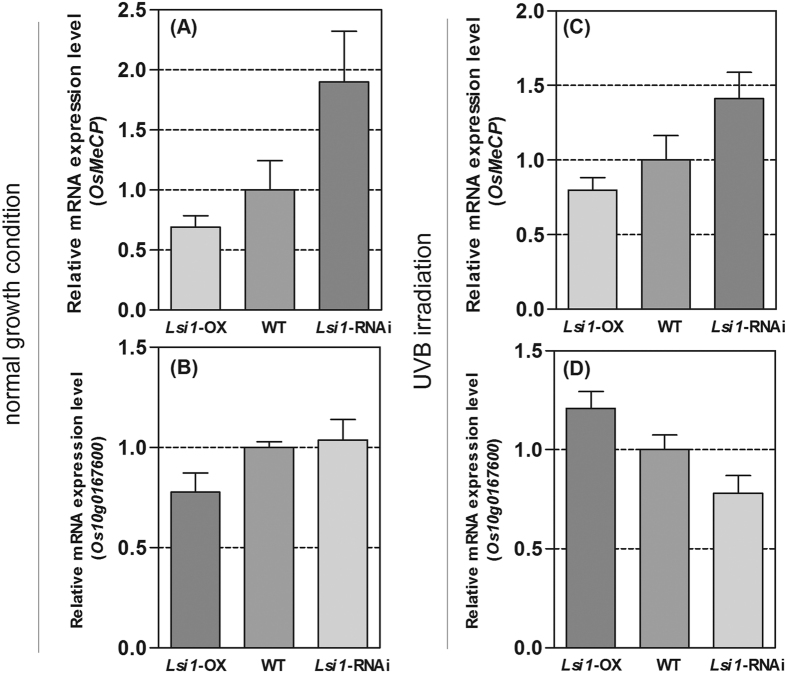
Changes in the gene expression levels of *OsMeCP* and *Os10g0167600* in *Lsi1*-OX, -RNAi, and WT. (**A**) mRNA expression of *OsMeCP* in the *Lsi1*-OX and -RNAi transformed Lemont lines compared to the WT under normal growth conditions. (**B**) mRNA expression of *Os10g0167600* in the *Lsi1*-OX and -RNAi transformed Lemont lines compared to the WT under normal growth conditions. (**C**) mRNA expression of *OsMeCP* in the *Lsi1*-OX and -RNAi transformed Lemont lines compared to the WT under UVB radiation for 5 h. (**D**) mRNA expression of *Os10g0167600* in the *Lsi1*-OX and -RNAi transformed Lemont lines compared to the WT under UVB radiation for 5 h. The gene expression levels in the WT were normalized as a value of 1. Expression level >1 and expression level <1 indicate up-regulation and down-regulation of gene expression in *Lsi1*-OX or -RNAi transformed rice compared to the WT under normal growth conditions and UVB radiation, respectively.

The gene transcription levels of *OsMeCP* were then detected in the three rice samples. *OsMeCP* was down-regulated in the *Lsi1*-OX rice compared with the WT, but was up-regulated in the *Lsi1*-RNAi line (Fig. 6A), suggesting that the silicon in the rice negatively regulated *OsMeCP* gene expression. Similarly, *Os10g0167600* gene expression was down-regulated in the *Lsi1*-OX rice and slightly up-regulated in the *Lsi1*-RNAi line, compared with the WT (Fig. 6B). This can be attributed to the low CPD content in the rice DNA under normal light condition (Fig. 1C). When the rice samples were exposed to UVB radiation, the *Lsi1*-OX rice still showed down-regulation of *OsMeCP* gene expression compared with the WT, and the reverse was true in the *Lsi1*-RNAi line (Fig. 6C). However, in contrast to *OsMeCP* gene expression in the rice samples, *Os10g0167600* gene expression was found to be up-regulated in the *Lsi1*-OX line but down-regulated in the *Lsi1*-RNAi line compared with the WT (Fig. 6D). Thus, it can be deduced that *Os*MeCP may have transcriptionally repressed *Os10g0167600* gene expression in the rice under UVB radiation.

### Protein expression of *Os*MeCP in the *Lsi1*-OX, -RNAi transformed Lemont, and WT lines

**Figure 7:**
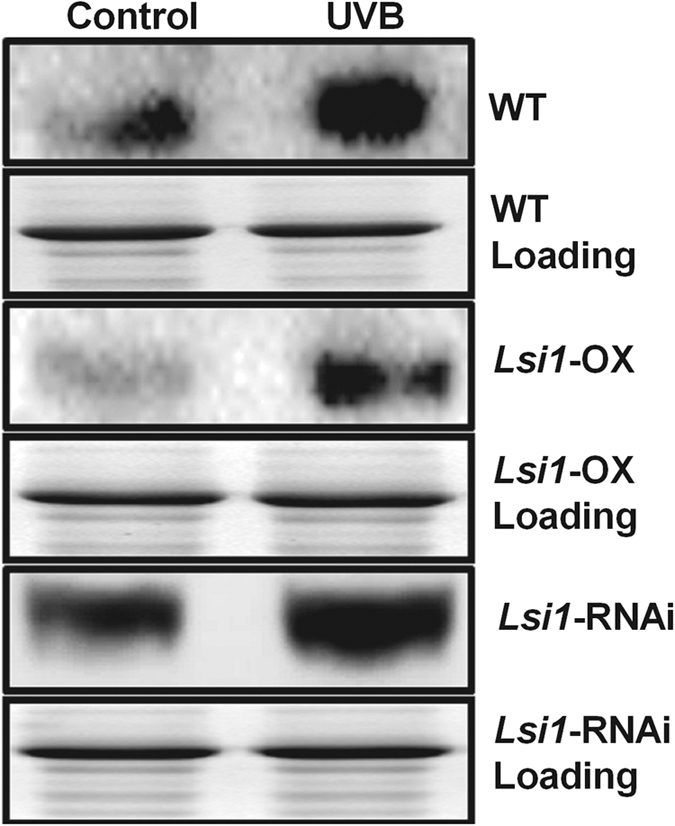
*Os*MeCP protein expression in the *Lsi1*-OX, -RNAi, and WT lines under UVB conditions detected by Western blot. “Control” indicates the rice in the control condition, with UVB and UVC eliminated by wrapping mylar film on the UV lamps; “UVB” indicates that lamps with a 0.1-mm film of cellulose diacetate were used as the source of UVB radiation. The lamps were applied at the top of the rice leaf canopy for 5 h.

The protein expression levels of *Os*MeCP were also detected in the transformed and WT rice lines under UVB radiation. It was found that the protein expression obviously increased in the rice exposed to UVB irradiation. The overexpression of *Lsi1* inhibited the expression of *Os*MeCP, and the reverse was true in the *Lsi1*-RNAi line from the control group and UVB-treated group conditions (Fig. 7). These results further confirmed that the silicon in the rice played a negative role in the regulation of *Os*MeCP protein expression.

### *Os*MeCP expression and CPD content in rice cultured with different silicon concentrations

**Figure 8:**
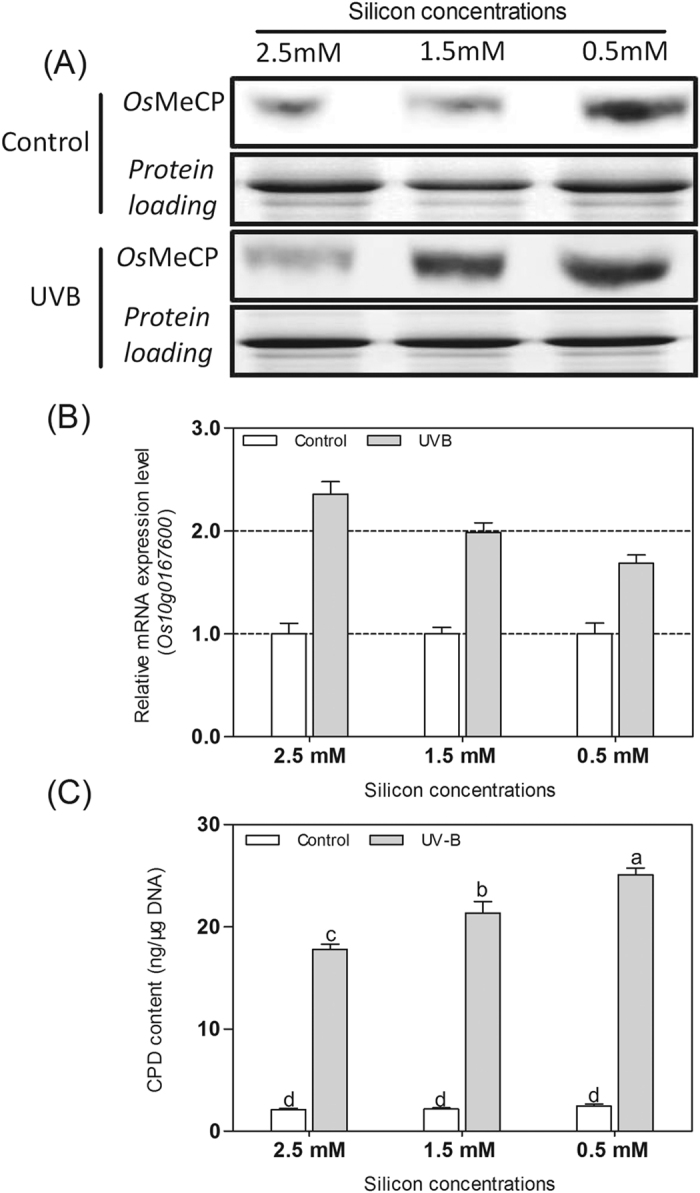
*Os*MeCP protein expression, *Os10g0167600* gene expression, and CPD content in the rice accessions cultured in hydroponic solution with different silicon concentrations. (**A**) *Os*MeCP protein expression levels in the UVB-treated rice cultured in hydroponic solutions containing 0.5 mM, 1.5 mM, and 2.5 mM of silicon, respectively, compared with the controls. (**B**) *Os10g0167600* gene expression levels in the UVB-treated rice samples cultured in hydroponic solutions containing 0.5 mM, 1.5 mM, and 2.5 mM silicon, respectively, compared with the controls. *Os10g0167600* gene expression levels in the WT control rice samples in hydroponic solution containing 0.5 mM, 1.5 mM, and 2.5 mM of silicon, normalized as a value of 1 to calculate the relative changes in expression compared with the UVB radiation condition. Expression level >1 and expression level <1 indicate up-regulation and down-regulation of the gene expression in the UVB treated group compared with the control group, respectively. (**C**) The CPD content in the UVB-treated rice samples compared with the controls. The different superscripts in the columns indicate statistical groups that are significantly different (p < 0.05) using Tukey’s range test.

Rice cultured in different silicate concentrations of the hydroponic solution showed different levels of *Os*MeCP. Increasing the silicate concentration in the solution resulted in reduced protein expression levels of *Os*MeCP in the rice. The protein expression level of *Os*MeCP was increased in the UVB-treated rice compared with the control group, where the two rice samples were under the same silicate concentration. Moreover, the protein level of *Os*MeCP was still the highest in the rice cultured in the medium containing 0.5 mM silicate, and lowest in that containing 2.5 mM of silicate (Fig. 8A). The gene expression level of *Os10g0167600* decreased slightly less in the UVB normal condition than in the rice cultured in the increasing silicate concentration. This may have been because the rice under the normal growth conditions had little DNA damage, as indicated by the lower CPD content on the rice leaves. When the three different cultured strains of rice were exposed to UVB radiation, the gene transcription of *Os10g0167600* increased in all cases and was highest in the 2.5 mM silicate cultured rice (Fig. 8B). Moreover, the CPD content was highest in the 0.5 mM silicate cultured rice, significantly decreased in the rice cultured with the hydroponic nutrient solution that contained 1.5 mM silicate, and was notably lowest in the 2.5 mM condition. Detection of the CPD content in the leaves of rice cultured in the different silicate concentrations showed that increasing the silicate concentration in the solution obviously repressed *Os*MeCP protein expression, and resulted in enhanced *Os10g0167600* gene expression in the UV-B treated rice, which strengthened the photoreactivation and significantly reduced the CPD content in the cultured rice leaves (Fig. 8C).

### *OsMeCP-*OX and -RNAi transformed rice and DNA damage

**Figure 9:**
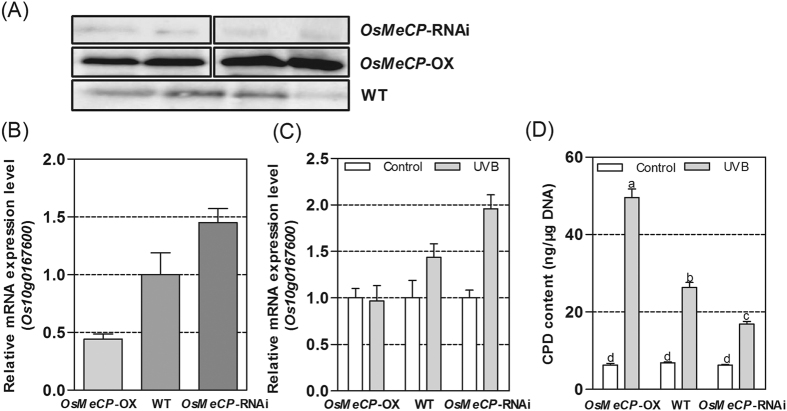
The CPD content in the *OsMeCP*-OX, -RNAi transformed Lemont, and WT lines under UVB radiation. (**A**) *Os*MeCP protein expression in the *OsMeCP*-OX, -RNAi transformed Lemont, and WT lines detected by Western blot. (**B**) mRNA expression of *Os10g0167600* in the *OsMeCP*-OX and -RNAi transformed Lemont lines compared with the WT under normal growth conditions. The gene expression level of *Os10g0167600* in the WT was normalized as a value of 1. Expression level >1 and expression level <1 indicate up-regulation and down-regulation of the gene expression in the transformed line compared to the WT, respectively. (**C**) mRNA expression level of *Os10g0167600* in the *OsMeCP*-OX and -RNAi transformed Lemont lines under UVB radiation compared with the control groups. The gene expression level of *Os10g0167600* in the control groups was normalized to a value of 1. Expression level >1 and expression level <1 indicate up-regulation and down-regulation of the gene expression in the UVB treated group compared with the control group, respectively. (**D**) CPD content in the three UVB-treated rice lines and their control groups. The different superscript letters in the columns indicate groups that are statistically significantly different (p < 0.05) in terms of the leaf CPD content of the *OsMeCP*-OX. -RNAi, and WT lines, as indicated by Tukey’s range test. *OsMeCP*-OX, OsMeCP overexpression transformed Lemont. *OsMeCP*-RNAi, *OsMeCP* RNA interference transformed Lemont.

The respective *OsMeCP*-OX and -RNAi transformed Lemont rice lines were generated. The protein expression level of *Os*MeCP in the overexpression transformed line was found to be greater than in the WT, and it was obviously reduced in the *OsMeCP*-RNAi line (Fig. 9A). Comparison of the changes in *Os10g0167600* gene expression in the two transformed rice lines and the WT line under normal growth conditions showed that *Os10g0167600* gene expression was up-regulated in the *OsMeCP*-RNAi line but down-regulated in the *OsMeCP*-OX line compared with the WT (Fig. 9B). When the three lines were exposed to UVB radiation, both *OsMeCP*-RNAi and the WT showed up-regulation of *Os10g0167600* gene expression compared with the control group, and the increase in the up-regulation was higher in the *OsMeCP*-RNAi transformed line than in the WT. In contrast, a slight down-regulation of *Os10g0167600* gene expression was found in the *OsMeCP*-OX transformed line (Fig. 9C). The highest up-regulation of *Os10g0167600* gene transcription in the *OsMeCP*-RNAi transformed line led to the lowest CPD content in the UVB-treated leaves, compared with that of the WT and the *OsMeCP*-OX transformed line. Moreover, the *OsMeCP*-OX transformed line had the highest CPD content among the three rice lines (Fig. 9D), owing to the slightly higher down-regulation of *Os10g0167600* than in the WT. These results further confirmed the transcriptional repression of *Os*MeCP on *Os10g0167600* gene expression under UVB radiation, which resulted in reduced photolyase activity in the UVB-exposed rice.

## Discussion

UVB radiation causes the formation of covalent links by reactions localized on the C=C double bonds, which results in the accumulation of CPDs and 6-4PPs in DNA^19^. To reduce the damage, the photoreactivation of the CPD photolyase repairs the CPD in the plant chloroplasts, mitochondria, and nuclei^20^, which is essential for plant survival after exposure to UVB-containing sunlight.

In rice plants, *Os10g0167600* encodes the deoxyribodipyrimidine photolyase, which is involved in the repair of UV radiation-induced DNA damage and catalyzes the light-dependent monomerization (300–600 nm) of CPDs, which is required for plant survival in the presence of UVB light. However, photolyase is not involved in the repair of 6-4 PPs^21^,^22^. In contrast to *Os10g0167600*, *Os02g0204400* encodes the (6-4) DNA photolyase that catalyzes the photoreactivation of 6-4 PPs. The Os03g0343400 protein is a putative photolyase/blue-light receptor PHR2, and Os09g0532700 is deoxyribodipyrimidine photolyase family protein-like, and both have hydrolase activity. Unlike the photolyase, *Os02g0625000*, *Os02g0573200*, and *Os06g0661800* encode cryptochromes with blue-light photoreceptor activity, but there is a lack of photolyase activity.

Although the transcript levels of these seven genes were detectable, we observed different expression levels in the three rice lines with different levels of *Lsi1* abundance. Among these genes, the transcription of *Os10g0167600* was correlated with the silicon content in the rice, and the highest increase was found in the *Lsi1*-OX transformed rice under UVB radiation. The resulting CPD content was negatively related to the gene abundance, and were lowest in the *Lsi1*-OX transformed rice. Such a reduction in the DNA lesion would maintain the UVB resistance of rice. Recently, Teranishi *et al.*^13^ found that CPD photolyase was overexpressed in both UVB-sensitive *O. sativa* Norin 1 (japonica), and UVB-hypersensitive *O. sativa* Surjamkhi (indica) when determining its function in relation to UVB resistance in rice. Their results showed that CPD photolyase is a vital factor for evaluating the UVB sensitivity of *O. sativa*^13^,^14^. Similar results were found in a study on *Arabidopsis thaliana*^23^,^24^.

Gene expression can be regulated by trans-acting factors, which are known as transcription factors. These regulators interact with the cis-regulatory elements to activate gene transcription. In our study, the *Os10g0167600* promoter was associated with the CpG islands, and *Os*MeCP was found to interact with the promoter to regulate the gene transcription. Protein subcellular locations are closely linked to their biological functions, and, in this study, the subcellular location of *Os*MeCP was found to be in the nucleus, which confirms its role in the regulation of transcription. A decrease in the protein abundance of *Os*MeCP in the rice cultured under the higher silicon condition indicates that silicon acts as a negative factor in the regulation of *Os*MeCP expression. The methyl-CpG-binding domain, which consists of about 70 residues, possesses a unique α/β-sandwich structure with characteristic loops, and is able to bind single methylated CpG pairs as a monomer, which is involved in recruiting histone deacetylases to methyl CpG-enriched regions in the genome to repress transcription^25^. We found that rice with a higher silicon content exhibited lower protein abundance of *Os*MeCP in both the *Lsi1*-OX transformed line and the WT cultured in high silicate-concentration solutions. This suggests that *Os*MeCP is a negative feedback of silicon. However, the changes in the gene transcription levels of *Os10g0167600* in the *Lsi1*-OX and *Lsi1*-RNAi transformed lines compared with the WT, and in the rice cultured in different silicon-concentration solutions, were opposite to the *Os*MeCP expression level in the same rice. This finding indicates the repression of *Os*MeCP in the transcription of *Os10g0167600* in the UVB-treated rice. Nan *et al.*^26^ documented that an abundant nuclear protein, the methyl-CpG-binding protein MeCP2, interacts specifically with methylated DNA and mediates the transcriptional repression. MeCP2 binds tightly to the chromosomes in a methylation-dependent manner and contains a transcriptional repression domain that can function at a distance *in vitro* and *in vivo*.

Overexpression of *OsMeCP* in rice also resulted in the down-regulation of *Os10g0167600* gene expression, while the gene transcription level of *Os10g0167600* was up-regulated in the *OsMeCP*-RNAi line. These results further clarify that *Os*MeCP acts as a negative regulatory factor in the transcription of *Os10g0167600*. The rice exposed to UVB radiation showed up-regulation of *Os10g0167600* gene expression, but it was still highest in the *OsMeCP*-RNAi line, which led to the lowest amount of CPDs on the DNA. In contrast, the highest CPD content was found in the *OsMeCP*-OX transformed line, which was attributable to the slight down-regulation of *Os10g0167600* gene expression under UVB treatment compared with the control group. Thus, the transcriptional repression on *Os10g0167600* was obviously mediated by OsMeCP.

**Figure 10:**
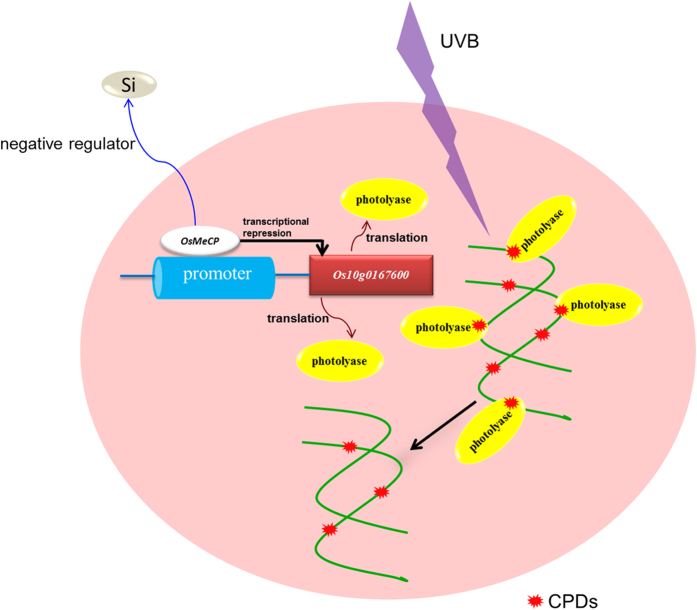
Schematic summary of the role of silicon in negatively regulating *Os*MeCP in rice to repress the transcriptional gene expression of *Os10g0167600* under UVB radiation, and of *Os10g0167600* in encoding the deoxyribodipyrimidine photolyase to repair the CPDs on rice DNA.

In conclusion, increasing the exogenous silicon concentration reduced the protein expression of *Os*MeCP in rice, and the silicon content in rice negatively regulated the protein expression of *Os*MeCP. The protein abundance of *Os*MeCP was also lower in the *Lsi1*-OX transformed line than in the WT rice, whereas the reverse was true for the *Lsi1*-RNAi line. *Os*MeCP repressed the transcription of *Os10g0167600* gene expression, leading to down-regulation of *Os10g0167600* in the rice samples. Inhibition of *OsMeCP* increased *Os10g016760* gene expression, which reduced the CPD content in the rice under UVB radiation, while overexpression of *OsMeCP* led to the reverse results. Overall, our results indicate that *Os*MeCP is a negative regulator of silicon uptake in rice and plays a role in repressing *Os10g0167600* gene transcription, which dominates the photorepair of damaged DNA in rice following UVB radiation (Fig. 10).

## Materials and Methods

### Plant materials

The Lemont rice accession (UVB-tolerant; introduced from the United States) was used in this study. The *Lsi1*-OX and *Lsi1*-RNAi transformed lines, which were generated in our previous study, had significantly higher or lower levels of silicon than the WT^18^.

The WT and transformed rice seeds were surface-sterilized with 25% NaClO for 30 min and then soaked in sterilized ddH_2_O overnight. The seeds were then placed in a temperature-controlled incubator to germinate at 30 °C, and the germinated seeds were sown in separate seedling plates. At the three-leaf stage, uniform seedlings from each plant were selected and transferred onto a Styrofoam plate (with holes spaced at 5 × 6 cm). The seedlings were affixed to the plate by inserting a cotton plug into each hole. The Styrofoam plate was allowed to float in a pot (45 × 35 × 15 cm) filled with 10 L of rice (*Oryza sativa* L.) culture solution (silicate-containing solution) with the following composition: 482 mg (NH_4_)_2_SO_4_, 248 mg KH_2_PO_4_, 185 mg KNO_3_, 149 mg K_2_SO_4_, 864.3 mg Ca(NO_3_)_2_.4H_2_O, 1350.6 mg MgSO_4_.7H_2_O, 2000 mg Na_2_SiO_3_.9H_2_O, 457 mg FeSO_4_.7H_2_O, 484.4 mg EDTA, 14.3 mg H_3_BO_3_, 0.4 mg CuSO_4_.5H_2_O, 1.1 mg ZnSO_4_.7H_2_O, 9.05 mg MnCl_2_.4H_2_O, and 0.45 mg Na_2_MoO_4_.2H_2_O. The solution was changed every week. The pot was continuously aerated, and its outer side was painted black to prevent algae growth. The pH value of the solution was maintained between 5.5 and 6.0 throughout the experiment. The method follows that of Fang *et al.*^18^, which is a minor optimization of the technique of Yoshida *et al.*^27^.

At the five-leaf stage, the transformed rice line and WT rice seedlings were exposed to enhanced UVB radiation following the method described by Fang *et al.*^18^,^28^. The procedure was as follows. Fluorescent lamps (40 W, Beijing Electric Light Sources Research Institute, China) were used as the source of UVB radiation. The lamps were suspended above the rice plants, and UV radiation of wavelengths less than 280 nm (i.e., UVC) was eliminated by wrapping the lamps in a 0.1-mm film of cellulose diacetate (West Design Product Co., Ltd, United Kingdom). The distance between the rice canopy and the lamps was 30 cm, and four lamps were used. UVB radiation was applied at the top of the leaf canopy from 10 am to 3 pm at an intensity of 18.6 kJ • m^−2^ • d^−1^. The rice seedlings used as a control were wrapped in a mylar film to filter UVB and UVC and placed under the lamps. The UVB-exposed and control rice leaves were sampled at 0.5, 1, 2, 3, and 5 h, and were immediately frozen in liquid nitrogen and stored at −80 °C for isolation of the total RNA to detect *Os10g0167600* gene expression. The sample of leaves treated for 5 h was also used to determine the silicon content and extract natural protein for the DNA-pull down.

### Detection of the rice silicon content

The fresh rice leaves were ground into powder in liquid nitrogen and oven-dried at 70 °C. Each 0.1 g portion of the dry powder was digested in 3 ml of 50% NaOH (W/V) in an autoclave sterilizer at 121 °C for 30 min. The mixture was centrifuged, and the supernatant liquor was diluted with ddH_2_O to 50 mL. One milliliter of the solution was added to 30 ml of 20% acetic acid (V/V) and 10 ml of ammonium molybdate solution (54 g/L; pH 7.0). The mixture was blended and allowed to sit for 5 min; 5 ml of 25% tartaric acid solution (W/V) and 1 ml of reducing agent was then added, and the mixture was finally diluted with 20% acetic acid to 50 ml. To prepare the reducing agent, 25 ml of ddH_2_O was dissolved with 2 g of Na_2_SO_3_ and 0.4 g of 1-amino-2-naphthol-4-sulfonic acid, and another 200 ml of ddH_2_O with 25 g NaHSO_3_ was combined and diluted with ddH_2_O to 250 mL.

The absorbance of the rice leaf solution was detected with a spectrophotometer at a wavelength of 650 nm. For the standard curve of the silicon content, a gradient of Na_2_SiO_3_ concentrations from 0, 1, 2, 3, 4, 5, 6, 7, 8, 9 mg/ml was taken to conduct the same reaction and then detected at OD_260_.

### Detection of CPDs and 6-4 PPs in transformed and WT rice

The DNA of the *Lsi1*-OX and *Lsi1*-RNAi transformed lines and the WT rice leaves were extracted with a NuClean PlantGen DNA Kit (Beijing ComWin Biotech Co. Ltd.), and the CPDs and 6-4 PPs on the DNA were detected with an OxiSelect™ UV-Induced DNA Damage ELISA Kit (CPD and 6-4 PP quantitation, respectively; Cell Biolabs, Inc., USA) following the manufacturers’ instructions.

### *Os10g0167600* gene expression in transformed and WT rice

The transcriptional abundance of the seven genes that encode photolyase or the photoreceptor was compared with our RNA-seq data to select the gene with the highest correlation with silicon. To detect the target *photolyase* gene expression in the UVB-irradiated *Lsi1*-OX and *Lsi1*-RNAi transformed lines and the WT, we designed gene-specific primers for *Os10g0167600* (see Table S1), and used a real-time quantitative polymerase chain reaction (qPCR) to detect the relative mRNA expression levels of the genes in the UVB-treated group and the control group. The reaction was performed in an Eppendorf realplex^4^ cycle, and the threshold cycle values (Ct) were recorded for each candidate mRNA in both the control and test samples. The 2^−△△Ct^ method was used to analyze the relative quantification of the gene expression^29^, and the gene expression level of *β-actin* was taken as a reference to normalize the candidate genes in the test sample.

### Cloning of the specific *photolyase* gene promoter and pulling the interacted proteins

Based on the comparative study of *photolyase* gene expression in the UVB-treated *Lsi1*-OX and -RNAi lines and the WT, *Os10g0167600* was selected as the available gene that was correlated with the silicon content of the rice strains and enhanced their capacity to repair the UVB damage on DNA. A 2134-bp upstream fragment of the 5′ end of the ORF of the gene was cloned and labeled with biotin (see Table S1). The natural proteins of the Lemont rice leaf were extracted with phosphate-buffered saline solution. The purified DNA fragment was mixed with the protein to reveal the interacted proteins. The DNA fragment was immobilized with streptavidin-coupled beads from the Dynabeads^®^ kilobaseBINDERTM Kit (Thermo Fisher Scientific Inc., USA), and the interacted proteins on the DNA fragment were then eluted with 1 M NaCl and forwarded to sodium dodecyl sulfate-polyacrylamide gel electrophoresis (SDS-PAGE). Liquid chromatography-mass spectrometry (LC-MS; LTQ, Thermo Scientific) was performed to identify the interacted proteins.

### Gene transcript levels of the DNA interacted proteins

A protein that contains the DNA binding domain, methyl-CpG binding domain protein (*Os*MeCP), was selected to detect the transcription levels in the *Lsi1*-OX, -RNAi transformed, and WT rice lines under UVB radiation for 5 h and the control. The specific primers for the encoding genes were as follows: *OsMeCP*-F: 5′-CTC ATC TGG CAG AAA GGG-3′ and *OsMeCP*-R: 5′-GGA AGG CTC AGT TGG GTT-3′. qPCR was used to detect the gene expression levels in the test rice.

### Protein expression detected by Western blot

To investigate the protein expression levels of *Os*MeCP in the different UVB-treated rice lines and the control we used monoclonal antibodies of *Os*MeCP (Abmart Inc., USA). The leaf proteins of the three UVB-treated rice samples and the control were extracted and separated by SDS-PAGE, and the monoclonal antibodies were found to be immune to the total protein. A Clarity Western ECL Kit (Bio-Rad Laboratories, Inc., USA) was used for the staining, and the protein expression level was checked with the ChemiDoc™ MP System (Bio-Rad Laboratories) under chemiluminescence.

### OsMeCP protein expression levels in rice cultured in different silicon concentrations of hydroponic solution

To detect the OsMeCP protein abundance, samples of the *Lsi1*-OX transformed Lemont rice were cultured in Yoshida culture solution with different silicate concentrations: 0.5 mM, 1.5 mM, and 2.5 mM. The rice under the three silicon concentrations was exposed to UVB radiation at the five-leaf stage, and rice wrapped in mylar film before being placed under the lamps was again used as the control. After 5 h, the respective rice leaves were sampled to detect OsMeCP protein abundance, *Os10g0167600* gene expression, and the CPD content.

### Protein subcellular localization of *Os*MeCP

The protoplast of rice was prepared to study the protein location. The full-length CDS of *OsMeCP* was amplified with gene-specific forward primer location CPG-F: 5′-ATC GC*T CTA GA*A TGG CCA CGG CCG GCG ACG A-3′ and location CPG-R: 5′-TAT AGG *ACT AGT*GGT GCA CTT CAC GGC AGA GG-3′ containing Xba I and Spe I sites (underlined). The gene was inserted into a plant binary expression vector, pCambia-eYFP-2300s, to create a recombinant plant transient expression vector and then transformed into the rice protoplast. The cell was cultured for two days to completely express the protein, and an Olympus Fluoview FV1000 laser scanning confocal microscope (Olympus, Tokyo, Japan) was used to observe the protein subcellular localization. The fluorescence images were acquired at 568-nm excitation and 592-nm emission. A monomeric far-red fluorescent protein TagFP635 (scientific name mKate) that tagged the nuclear localization signal protein localized to the nucleus was taken as the standard marker.

### Overexpression and RNA interference of *OsMeCP* in rice

To further confirm the gene function of *OsMeCP*, the full-length CDS of the gene was again amplified with primers containing different recognition sites for Sac I and Bam H I, respectively, in forward primer *OsMeCP*-*OX*-F: 5′-TAT AC*G AGC TC*A TCC CCA AAT CCC CAC ACG TC-3′ and reverse primer *OsMeCP*-*OX*-R: 5′-TAG CG*G GAT CC*C CAG CAA CGT CAG TTC CTT GG-3′. The gene was then inserted into another plant binary expression vector, pCambia-1300. A 347 base pair (bp) partial coding region downstream of the ATG of rice *OsMeCP* was cloned with a forward primer 5′-CG*GGATCC*AAATGAAGAAGC GAAAGACG-3′ and a reverse primer 5′-GG*GGTACC*AAGGCTCAGTTGGGTTGC-3′ containing *BamH I* and *Kpn I* sites (underlined), respectively. The same fragment was again amplified with the primers containing different recognition sites for *Sac I* and *Spe I*, respectively, in forward primer 5′-C*GAGCTC*AAATGAAGAAGCGAAAGACG-3′ and reverse primer 5′-GG*ACTAGT*AAG GCTCAGTTGGGTTGC-3′. Both fragments were inserted into pTCK303 to create an *OsMeCP*-RNAi stability vector. The two recombinant vectors were then transformed into the Agrobacterium strain EHA105, and the process of rice genetic transformation followed the methods described by Fang *et al.*^18^. After the positively transformed rice was confirmed and selected, the transformed rice and the WT were exposed to UVB radiation for 5 h, and the second top leaves of the rice plants were sampled to extract DNA and RNA, respectively, to detect the CPD contents and *Os10g0167600* gene expression in the different types of rice under UVB irradiation.

## Additional Information

**How to cite this article**: Fang, C. *et al.* Methyl-CpG binding domain protein acts to regulate the repair of cyclobutane pyrimidine dimers on rice DNA. *Sci. Rep.* **6**, 34569; doi: 10.1038/srep34569 (2016).

## Supplementary Material

Supplementary Information

## Figures and Tables

**Table 1:** The target protein interacted with promoter of *Os10g0167600* identified by LC-MS.

Accession	Coverage	# PSMs	# Peptides	# AAs	MW [kDa]	calc. pI	Score	Description
LOC_Os12g42550.2 protein	33.78	10	5	299	31.0	4.79	54.38	methyl-CpG binding domain containing protein, putative, expressed
