# Supplementary material for: Methyl-CpG binding domain protein acts to regulate the repair of cyclobutane pyrimidine dimers on rice DNA
Source: Sci Rep. 2016 Oct 3;6:34569. doi: 10.1038/srep34569 (PMC5046113; doi:10.1038/srep34569)
Supplement: Supplementary Information [file srep34569-s1.pdf]

Title: Methyl-CpG binding domain protein acts to regulate the repair of cyclobutane pyrimidine dimers on rice DNA

Author names: Changxun Fang, Weisi Chen, Chengxun Li, Xin Jian, Yingzhe Li, Hongmei Lin, Wenxiong Lin

Supplementary Tables

Table S1 The primers used in this study

Gene	Forward primer	Reverse primer	Aim
Os10g0167600	5'-GCTGCTGATAAAAGAGAG-3'	5'-CACATACATCCGACGTAA-3'	qPCR
OsMeCP			qPCR
Actin	5’-CTGCGGGTATCCATGAGACT-3’	5’-GCAATGCCAGGGAACATAGT-3’	qPCR
Os10g0167600	5'-CATGTAGAGCTTATCCCTACAGGCC-3' (biotin was labeled at 5’ end)	5’-CCCATGCACCAGACAGCGAATCATA -3’	Promoter
OsMeCP-RNAi-F	5' CGGGATCCAAATGAAGAAGCGAAAGACG 3'(BamH I sites underlined)	5' GGGGTACCAAGGCTCAGTTGGGTTGC 3'(Kpn I sites underlined)	RNAi
OsMeCP-RNAi-R	5' GGACTAGTAAGGCTCAGTTGGGTTGC 3'(Spe I sites underlined)	5' CGAGCTCAAATGAAGAAGCGAAAGACG 3'(Sac I sites underlined)	RNAi
OsMeCP-OX	5'-TATACGAGCTCATCCCCAAATCCCCACACGTC-3'(Sac I sites underlined)	5'-TAGCGGGATCCCCAGCAACGTCAGTTCCTTGG-3'(BamH I sites underlined)	Overexpression

Table S2 The putative proteins interacted with promoter of *Os10g0167600* identified by LC-MS

Accession	Coverage	PSMs	Peptides	AAs	MW [kDa]	calc. pI	Score	Description
LOC_Os08g33370.2 protein	30.08	9	6	256	28.8	4.84	39.44	14-3-3 protein, putative, expressed
LOC_Os02g32030.1 protein	5.93	5	3	843	94.0	6.16	35.13	Elongation factor, putative, expressed
LOC_Os03g19410.1 protein	3.62	3	2	938	103.8	5.95	19.82	*Os*POP7 - Putative Prolyl Oligopeptidase homologue, expressed
LOC_Os01g53900.1 protein	4.12	3	2	826	91.9	6.76	18.47	Elongation factor, putative, expressed
LOC_Os04g38600.3 protein	10.70	5	3	402	42.7	7.72	40.13	Glyceraldehyde-3-phosphate dehydrogenase, putative, expressed
LOC_Os11g02450.1 protein	9.17	2	2	229	25.1	9.03	35.67	Elongation factor P, putative, expressed
LOC_Os12g42550.2 protein	33.78	10	5	299	31.0	4.79	54.38	Methyl-CpG binding domain containing protein, putative, expressed
LOC_Os03g63480.1 protein	9.71	4	2	350	37.2	4.70	27.54	Ankyrin repeat domain containing protein, expressed
LOC_Os02g41590.1 protein	9.68	3	2	341	37.0	5.16	20.14	Kinase, pfkB family, putative, expressed
